# The Clinicopathological Characteristics of Alpha-Fetoprotein-Producing Adenocarcinoma of the Gastrointestinal Tract—A Single-Center Retrospective Study

**DOI:** 10.3389/fonc.2021.635537

**Published:** 2021-04-29

**Authors:** Xiang-Xing Kong, Xin-Lin Li, Yu Tian, Qian-Cheng Ye, Xiao-Ming Xu, Yue Liu, Qi Yang, Li-Na Zhang, Yan-Xia Mei, Ji-Hang Wen, Qian Xiao, Jing-Song Li, Ke-Feng Ding, Jun Li

**Affiliations:** ^1^Department of Colorectal Surgery and Oncology, Key Laboratory of Cancer Prevention and Intervention, Ministry of Education, The Second Affiliated Hospital, Zhejiang University School of Medicine, Hangzhou, China; ^2^Zhejiang University Cancer Center, Hangzhou, China; ^3^Department of Breast Surgery, Hwa Mei Hospital, University of Chinese Academy of Sciences, Ningbo, China; ^4^Engineering Research Center of EMR and Intelligent Expert System, Ministry of Education, College of Biomedical Engineering and Instrument Science, Zhejiang University, Hangzhou, China; ^5^Department of Pathology, Key Laboratory of Cancer Prevention and Intervention, Ministry of Education, The Second Affiliated Hospital, Zhejiang University School of Medicine, Hangzhou, China; ^6^Research Center for Healthcare Data Science, Zhejiang Lab, Hangzhou, China

**Keywords:** AFP-producing, gastrointestinal, adenocarcinoma, clinical characteristic, pathological characteristic

## Abstract

Alpha-fetoprotein (AFP)-producing adenocarcinoma from the gastrointestinal tract (APA-GI) is a rare type of highly malignant tumor with a poor prognosis. It may originate from any site along the GI tract with similar clinicopathological characteristics. As limited research had ever described the characteristics of APA-GI, the present article intends to systemically investigate the clinicopathological characteristics of APA-GI from a single center's retrospective study to deepen the understanding of the disease. A total of 177 patients pathologically diagnosed with APA-GI between 2010 and 2017 at the Second Affiliated Hospital of Zhejiang University, School of Medicine, were included. Also, clinical data of 419 gastric cancers and 609 colorectal cancers from The Cancer Genome Atlas database were also extracted. Clinical information of patients from Second Affiliated Hospital of Zhejiang University, School of Medicine, was collected, and a median follow-up of 14.5 months was performed to investigate clinical characteristics of APA-GI. For the pathological characteristics of APA-GI, hematoxylin–eosin sections were reviewed, and immunohistochemistry of AFP was performed. The results showed that the primary tumor could develop through the whole GI tract, including the esophagus (0.6%), stomach (83.1%), duodenum (1.1%), ileum (0.6%), appendix (0.6%), colon (5.1%), and rectum (7.9%). Hepatoid adenocarcinoma is the main pathological feature of APA-GI. AFP expression level in tumor tissue was not strictly associated with serum AFP or hepatoid differentiation. The prognosis of APA-GI was worse than that of common adenocarcinoma of the GI tract and liver metastasis, and high AFP levels suggest poor prognosis in patients with APA-GI. Therefore, the present study was the first research to systemically explore the clinicopathological characteristics of APA-GI. APA-GI occurs through the whole GI tract with a significantly worse prognosis than common adenocarcinoma of GI. APA-GI should be regarded as one kind of disease for its similar clinicopathological characteristics within patients.

## Introduction

Alpha-fetoprotein (AFP) is a glycoprotein produced by the fetal liver, yolk sac, and fetal gastrointestinal cells. Its content in adult serum is extremely low, and its abnormal increase is mainly found in hepatocellular carcinoma and yolk sac-derived tumors (1). However, abnormally elevated serum AFP is also reported in some kinds of adenocarcinoma, which are called AFP-producing adenocarcinoma (APA) (2). APA is a rare type of highly malignant tumor with a fairly poor prognosis (3, 4). It can originate from a variety of organs and mainly occurs in the digestive tract. The majority of APAs are developed in the stomach, accounting for 1.3–15% of all gastric cancers (GC) and <1% of all colorectal cancers (CRCs) (5, 6). However, much less is known about the clinicopathological characteristics of APA.

The serum AFP in APA patients is increased (>20ng/ml) and markers representing embryonic stem cells and hepatocellular carcinoma in tumor tissues, such as AFP protein, glypican-3, Sal-like protein-4, and/or hepatocyte antigen-1, are positive (7, 8). APA originated from the gastrointestinal tract (APA-GI) was reported to have some common clinical characteristics, such as early multi-organ metastasis, multidrug resistance, rapidly worsen after diagnosis, and much inferior prognosis than common adenocarcinoma of the gastrointestinal tract (CA-GI) (5, 6, 9–11). Although the previous studies of APA-GI usually only included APA-GC or APA-CRC (3, 12), we found APA-GI could also originate from the whole GI tract, including the esophagus, small intestinal, and appendix.

Therefore, we believe that APA-GI is a separate class of disease that showed similar clinicopathological characteristics within different origins. As the research on APA-GI is limited, how to accurately differentiate it from CA-GI and get accurate treatment to improve the prognosis is still in challenge (2, 13). The present article intends to systemically investigate the clinical and pathological characteristics of APA-GI from a single center's retrospective study to deepen the understanding of the disease.

## Patients and Methods

### Patients From the Second Affiliated Hospital of Zhejiang University, School of Medicine

Patients pathologically diagnosed with APA-GI between 2010 and 2017 at the Second Affiliated Hospital of Zhejiang University, School of Medicine (SAHZU) were included in the study. Also, the exclusion criteria were the following: (1) pregnancy status; (2) synchronously diagnosed with acute or chronic hepatitis, liver cirrhosis, hepatocellular carcinoma, teratoma, and germinoma, or (3) cancer history other than GI adenocarcinoma. APA-GI was defined as follows: (1) pathologically confirmed primary gastrointestinal adenocarcinoma and (2) serum AFP>20 ng/ml during the whole course. Two pathologists reviewed all hematoxylin–eosin (HE) sections to exclude the yolk sac tumor-like area or neuroendocrine component.

### Patients From the Cancer Genome Atlas

A total of 419 GC patients and 609 CRC patients from The Cancer Genome Atlas (TCGA) database (URL: http://cancergenome.nih.gov) were included. All these patients have pathologically confirmed adenocarcinoma.

### Data Collection and Follow-Up

For patients from SAHZU, clinical characteristics were all collected from the hospital information system, which included age, sex, primary tumor location, tumor size, pathological type, differentiated level, tumor–node–metastasis (TNM) staging, metastasis status, operation method, operation time, post operation pathology, serum AFP level, and treatment strategy. The last follow-up date was January 1, 2019. The major approach for follow-up was telephone calls or outpatient visits.

For patients from TCGA, we extracted patients' sex, pathology, TNM staging, survival time, survival status, and *via* R.

### Immunohistochemistry

Immunohistochemistry (IHC) staining was performed according to the standard protocol. Freshly cut 4-μm paraffin-embedded sections were incubated overnight at 62°C and then de-paraffinized by xylene and dehydrated with ethanol. Antigen retrieval was performed using a pressure cooker in citrate antigen retrieval solution (pH 8.0) for 15 min, then incubated with 3% peroxide for 10 min and blocked by nonspecific staining blocking reagent (Dako, Glostrup, Denmark). Staining was performed with AFP polyclonal antibody (ab169552, Abcam). Briefly, 150 μl of rabbit polyclonal anti-AFP antibody at 100× dilution was incubated for 120 min, washed, subsequently incubated with horseradish peroxidase and anti-rabbit antibody-conjugated polymer for 30 min, washed, and finally incubated with 3,3'diaminobenzidine substrate for signal development. Sections were at last counterstained with hematoxylin.

Two pathologists independently reviewed the IHC sections. The staining intensity was scored as 0 (negative), 1 (weak), 2 (moderate), or 3 (strong), whereas the staining extent was scored as 0 (<5 %), 1 (5–25 %), 2 (26–50 %), 3 (51–75 %), and 4 (>75 %) according to the positive staining area proportion. Scores for staining intensity and extent were then multiplied to generate the immunoreactivity score (IRS) for each case. Therefore, IRSs 0–1, 2–3, 4–8, and 9–12 were referred to negative, week positive, moderately positive, and strongly positive, respectively. IRS <4 was termed as AFP low expression, whereas IRS ≥ 4 was termed as AFP high expression.

### Statistical Analysis

The chi-squared test and Fisher's exact analysis were performed to compare the clinical and pathological characteristics between groups. Non-Gaussian-distributed data were presented as medians and interquartile ranges and evaluated by the Kruskal–Wallis test. Survival data were presented by Kaplan–Meier survival curves and compared using the log-rank test. With the Cox proportional hazards model, univariate and multivariate survival analyses were conducted to identify independently significant variables. Multivariate analyses were all based on factors <0.5, which were tested by univariate analyses. A *P*-value of 0.05 or less indicated statistical significance. Statistical and graphical analyses were performed with SPSS 23.0 and GraphPad Prism 7 software.

## Results

### Clinical Characteristics of Alpha-Fetoprotein-Producing Adenocarcinoma From the Gastrointestinal Tract

A total of 177 APA-GI patients were finally included. The median age was 63 years old (interquartile range, 53.5–71.0 years old), and males accounted for 69.5%. The primary tumor could develop through the whole gastrointestinal tract, including the esophagus (0.6%), stomach (83.1%), duodenum (1.1%), ileum (0.6%), appendix (0.6%), colon (5.1%), and rectum (7.9%). Meanwhile, it could also synchronously develop in two organs, such as the stomach and rectum (0.6%) and colon and rectum (0.6%). The pathological type of APA-GI included adenocarcinoma with high-, media-, and low-differentiated, signet-ring cell carcinoma and mucinous adenocarcinoma. Among 71 non-metastasis patients, 18.3% received operation, whereas 77.5% received a combination of treatments based on surgery. Within all APA-GI patients, 58.2% of patients present metastasis at the initial diagnosis, whereas 145 (81.9%) of patients finally developed metastasis. The liver was the most common site of APA-GI metastasis with a rate of 53.1%. Among gastric and colorectal APAs, 71 (40.1%) and 11 (47.8%) of patients were initially diagnosed with liver metastasis, respectively (Table 1).

**Table 1:** Baseline Characteristics of APA-GI.

**Characteristic**	**Total**
Age(y), median(IQR)	63.0 (53.5-71.0)
Sex, Male/Female (Male%)	123/54 (69.5)
**Primary site**, ***n*** **(%)**	
Stomach	147 (83.1)
Colon	9 (5.1)
Rectum	14 (7.9)
Appendix	1 (0.6)
Duodenum	2 (1.1)
Ileum	1 (0.6)
Esophagus	1 (0.6)
Stomach and rectum	1 (0.6)
Colon and rectum	1 (0.6)
Tumor size(cm), median(IQR)	4.5 (3.1-5.5)
Pathological type, *n* (%)	
Adenocarcinoma	147 (83.1)
Signet ring cell carcinoma	20 (11.3)
Mucinous adenocarcinoma	10 (5.6)
**Differentiation**, ***n*** **(%)**	
Highly differentiated	3 (1.7)
Moderately differentiated	43 (24.3)
Poorly differentiated	109 (61.6)
Default	22 (12.4)
**pT**, ***n*** **(%)**	
1	8 (8.2)
2	12 (12.4)
3	12 (12.4)
4	58 (59.8)
Default	7 (7.2)
**pN**, ***n*** **(%)**	
0	20 (20.6)
1	17 (17.5)
2-3	50 (51.5)
Default	10 (10.3)
**TNM staging**, ***n*** **(%)**	
I	8 (4.5)
II	17 (9.6)
III	46 (26.0)
IV	103 (58.2)
Default	3 (1.7)
**Metastatic site**^**#**^, ***n*** **(%)**	
Liver	94 (53.1)
Lymph node	71 (40.1)
Lung	19 (10.7)
Peritoneal cavity	16 (9.0)
Bone	15 (8.5)
Adrenal gland	7 (4.0)
Pelvic cavity	4 (2.3)
Spleen	4 (2.3)
Pancreas	4 (2.3)
Ovary and fallopian tube	4 (2.3)
Brain	3 (1.7)
**Lymphatic or/and blood vessel invasion**	
Yes	39 (40.2)
No	38 (39.2)
Default	20 (20.6)
**Nerve invasion**	
Yes	26 (26.8)
No	54 (55.7)
Default	17 (17.5)
Hepatoid Adenocarcinoma*	
Yes	34 (34%)
No	66 (66%)
**Treatments**	
Chemotherapy	46 (26.0)
Surgery	17 (9.6)
Comprehensive treatment	90 (50.8)
Other(Symptomatic treatment?Chinese herbology)	24 (13.5)

# For patients with multiple metastases, all the metastatic foci of different parts were included separately; *, Only 100 patients receiving second pathological diagnosis were included.

Increased serum AFP characterizes APA-GI. We found the AFP-high group (AFP-H group, AFP ≥ 200 ng/ml) had more stage IV (*P* = 0.009) and liver metastases (*P* < 0.001) when compared with the AFP-low group (AFP-L group, AFP <200 ng/ml). Additionally, the AFP-H group was characterized by larger tumor size (*P* = 0.047), a higher proportion of adenocarcinoma (*P* = 0.022), and hepatoid adenocarcinoma (HA) (*P* = 0.006) (Table 2).

**Table 2:** Comparison of clinical baseline characteristics of APA-GI patients with different serum AFP levels.

**Characteristic**	**AFP-L**	**AFP-H**	***P* value**
Age(y), median(IQR)	63.0 (52.0-72.0)	63.0 (57.0-69.0)	0.814
Sex, Male/Female (Male%)	73/35 (67.6)	50/19 (72.5)	0.492
Primary site, *n* (%)			0.596
Stomach	88 (81.5)	59 (85.5)	
Large intestine	17 (15.7)	7 (10.1)	
Small intestine	1 (0.9)	2 (2.9)	
Esophagus	1 (0.9)	0 (0)	
Multiple primary sites	1 (0.9)	1 (1.4)	
Tumor size(cm), median(IQR)	4.0 (3.0-5.0)	5.0 (4.0-6.0)	0.047
Pathological type, *n* (%)			0.022
Adenocarcinoma	83 (76.9)	64 (92.8)	
Signet ring cell carcinoma	17 (15.7)	3 (4.3)	
Mucinous adenocarcinoma	8 (7.4)	2 (2.9)	
Differentiation, *n* (%)			0.473
Highly differentiated	1 (0.9)	2 (2.9)	
Moderately differentiated	27 (25.0)	16 (23.2)	
Poorly differentiated	64 (59.3)	45 (65.2)	
Default	16 (14.8)	6 (8.7)	
pT, *n* (%)			0.524
1	7 (10.6)	1 (3.2)	
2	10 (15.2)	2 (6.5)	
3	8 (12.1)	4 (12.9)	
4	37 (56.1)	22 (71.0)	
Default	4 (6.1)	2 (6.5)	
pN, *n* (%)			0.643
0	12 (18.2)	8 (25.8)	
1	13 (19.7)	4 (12.9)	
2-3	33 (50.0)	17 (54.8)	
Default	8 (12.1)	2 (6.5)	
TNM staging, *n* (%)			0.009
I-III	51 (47.2)	20 (29.0)	
IV	54 (50.0)	49 (71.0)	
Default	3 (2.8)	0 (0.0)	
Liver metastasis			<0.001
Yes	41 (38.0)	53 (76.8)	
No	67 (62.0)	16 (23.2)	
Extrahepatic metastasis			0.962
Yes	63 (58.3)	40 (58.0)	
No	45 (41.7)	29 (42.1)	
Lymphatic or/and blood vessel invasion			0.509
Yes	24 (36.4)	15 (48.4)	
No	28 (42.4)	10 (32.3)	
Default	14 (21.2)	6 (19.4)	
Nerve invasion			0.126
Yes	14 (21.2)	12 (38.7)	
No	38 (57.6)	16 (51.6)	
Default	14 (21.2)	3 (9.7)	
Hepatoid Adenocarcinoma^#^			0.006
Yes	16 (32.7)	16 (66.7)	
No	33 (67.3)	8 (33.3)	

# *Considering the lack of pathological sample obtained from endoscopy, 73 surgical patients out of 100 patients receiving second pathological diagnosis were included*.

### Hepatoid Adenocarcinoma Is the Main Pathological Feature of Alpha-Fetoprotein-Producing Adenocarcinoma From the Gastrointestinal Tract

**Figure 1:**
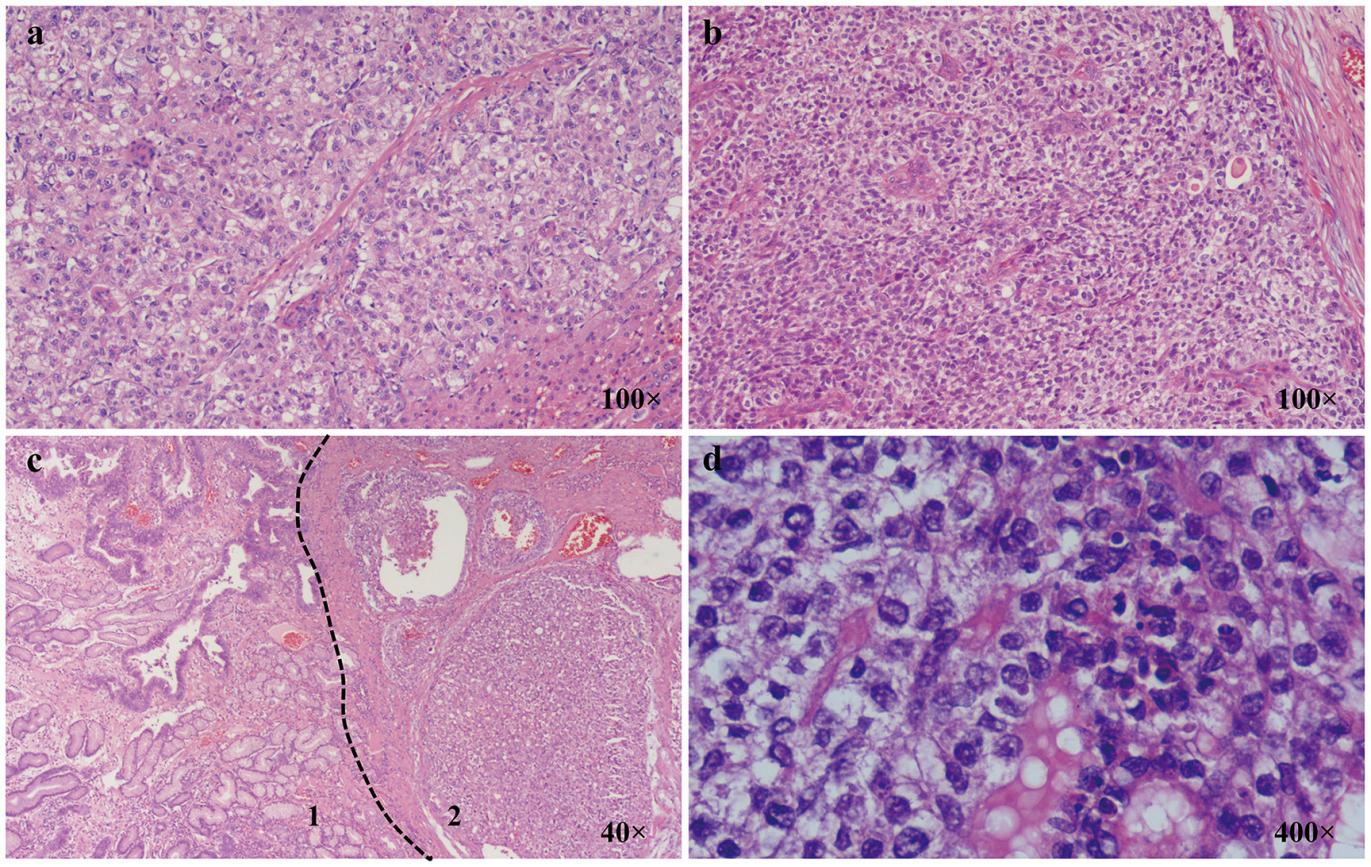
Characteristics of AFP-GI under a light microscope. **(a)** Classic primary hepatic carcinoma (100×); **(b)** Classic hepatoid adenocarcinoma (100×); **(c)** 1. Zone of adenocarcinoma with moderately differentiation; 2. Zone of adenocarcinoma with hepatoid differentiation (40×); **(d)** Zone of adenocarcinoma with hepatoid differentiation is composed of cells with round nuclei, coarse chromatin, obvious nucleoli, and clear cytoplasm (400×).

Two pathologists reviewed the 100 HE sections available. Thirty-four sections were reported (32 surgical samples and 2 endoscopic samples) containing HA regions. However, only three of these were first diagnosed as HA. Similar to primary hepatocellular carcinoma, HA consists of hepatoid cells with abundant blood sinuses. Cancer cells from HA often present with round nuclei, hyper-chromatin, prominent nucleoli, and clear cytoplasm. HA is also usually nested or banded and can be easily confused with normal adenocarcinoma (Figure 1).

We further compared the clinical characteristics between HA and non-HA within APA-GI patients. Seventy-three patients who received the operation were included. Compared with the non-HA group (41 cases), the HA group (32 cases) had more male patients (*P* = 0.002) and a higher proportion of low-differentiation (*P* = 0.007) (Table 3). However, the 5-year survival was insignificant between these two groups (non-HA and HA groups: 46.9 vs. 18.5%, respectively, *P* = 0.0953, Supplementary Figure 1).

**Table 3:** Comparison of clinical baseline characteristics between APA-GI patients with HA and non-HA.

**Characteristic**	**non-HA**	**HA**	***P*-value**
Age(y), median(IQR)	61.0 (50.0–70.5)	63.0 (58.0–71.8)	0.247
Sex, Male/Female (Male%)	22/19 (53.7)	28/4 (87.5)	0.002
Primary site, *n* (%)			0.321
Stomach	33 (80.5)	27 (84.4)	
Large intestine	7 (17.1)	3 (9.4)	
Small intestine	1 (2.4)	0 (0.0)	
Esophagus	0 (0.0)	1 (3.1)	
Multiple primary sites	0 (0.0)	1 (3.1)	
Tumor size(cm), median(IQR)	4.5 (3.5–5.3)	5.0 (3.0–6.0)	0.668
Differentiation, *n* (%)			0.007
Highly differentiated	0 (0.0)	0 (0.0)	
Moderately differentiated	17 (41.5)	5 (15.6)	
Poorly differentiated	21 (51.2)	27 (84.4)	
Default	3 (7.3)	0 (0.0)	
pT, *n* (%)			0.913
1	3 (7.3)	2 (6.3)	
2	6 (14.6)	4 (12.5)	
3	5 (12.2)	6 (18.8)	
4	27 (65.9)	20 (62.5)	
pN, *n* (%)			0.825
0	8 (19.5)	7 (21.9)	
1	6 (14.6)	7 (21.9)	
2-3	25 (61.0)	17 (53.1)	
Default	2 (4.9)	1 (3.1)	
TNM staging, *n* (%)			0.514
I-III	31 (75.6)	22 (68.8)	
IV	10 (24.4)	10 (31.3)	
Liver metastasis			0.084
Yes	9 (22.0)	13 (40.6)	
No	32 (78.0)	19 (59.4)	
Extrahepatic metastasis			0.756
Yes	22 (53.7)	16 (50.0)	
No	19 (46.3)	16 (50.0)	
Lymphatic or/and blood vessel invasion			0.269
Yes	19 (46.3)	19 (59.4)	
No	22 (53.7)	13 (40.6)	
Nerve invasion			0.486
Yes	11 (26.8)	11 (34.4)	
No	30 (73.2)	21 (65.6)	

*HA, hepatic adenocarcinoma; non-HA, non-hepatic adenocarcinoma*.

### Alpha-Fetoprotein-Producing Was Not Strictly Expressed in Alpha-Fetoprotein-Producing Adenocarcinoma From the Gastrointestinal Tract Patients' Tumor Tissue

IHC was performed in 99 tumor specimens to explore the AFP expression in APA-GI patients. IRS was then evaluated by two pathologists independently (Supplementary Figure 2). There were 15 AFP high expression patients, and the other 84 patients were classified as AFP low expression. AFP was positive in 36.4% of APA-GI patients, and it was also positive in 43.3% of patients with elevated serum AFP before surgery. Interestingly, AFP was only partly expressed in HA with a positive rate of 73.5%. Among the 36 patients with positive IHC AFP, 5.6% had normal levels of AFP before surgery. Therefore, the AFP expression level in tumor tissue was not strictly associated with serum AFP or hepatoid differentiation. Additionally, for 73 patients who received the operation, 35 patients had neither positive AFP IHC nor HA (AFP-/HA-), whereas the others performed at least one characteristic. However, the 5-year survival between these two groups was similar (44.54 vs. 24.26%, *P* = 0.342, Supplementary Figure 3).

### Prognosis of Alpha-Fetoprotein-Producing Adenocarcinoma From the Gastrointestinal Tract Was Worse Than That of Common Adenocarcinoma of the Gastrointestinal Tract

To investigate the prognosis of APA-GI, a median follow-up of 14.5 months was performed. There were only 31 (17.5%) who survived, whereas 129 (72.9%) died and 17 (9.6%) lost follow-up among the patients included. The 1-, 3-, and 5-year overall survival rates were 50.6, 28.5, and 17.7%, respectively.

**Figure 2:**
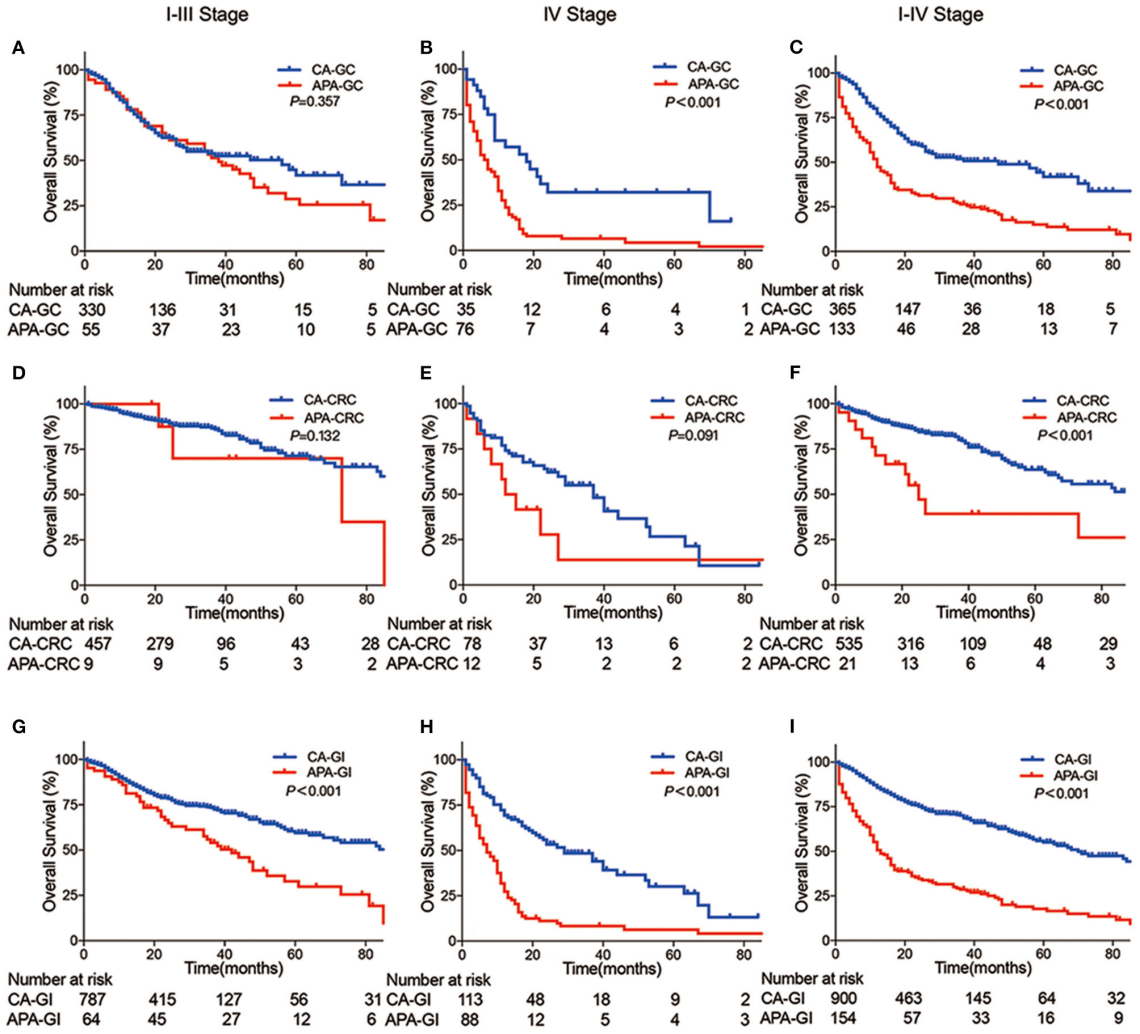
Kaplan–Meier curve between APA-GI and CA-GI. **(A–C)** Survival differences between APA-GC and CA-GC; **(D–F)** survival differences between APA-CRC and CA-CRC; **(G–I)** survival differences between APA-GI and CA-GI; Column 1 shows survival comparison between stages I–III patients. Column 2 shows survival comparison between stage IV patients. Column 3 shows survival comparison between stages I–IV patients. APA-GI patients were composed of 154 APA-GC and APA-CRC from the SAHZU database, whereas CA-GI patients were all extracted from TCGA database. Notably, two APA-GC patients were unable to perform accurate staging due to the lack of medical imaging data. Therefore, they can only be classified as stages I–IV.

To further compare the prognosis between APA-GI and CA, we extracted the follow-up information of CA-GC and CA-CRC from TCGA. For this reason, only 154 APA-GC and APA-CRC patients of 177 APA-GI patients from our center were analyzed. There was no significant difference in the 5-year survival between stages I–III APA-GC and CA-GC (28.8 vs. 41.9%, respectively, *P* = 0.357). However, the prognosis of APA-GC in stage IV was significantly worse than that of CA-GC (4.4 vs. 32.1%, *P* < 0.001). The 5-year survival of patients with APA-CRC was similar to CA-CRC (5-year survival: stages I–III APA-CRC and CA-CRC, 71.32 vs. 70%, respectively, *P* = 0.132; stage IV APA-CRC and CA-CRC, 13.89 vs. 26.73%, respectively, *P* = 0.091). After combining CRC and GC, survival analysis of APA-GI from our center showed significantly worse 5-year survival than that of CA-GI from TCGA (stages I–III, 32.8 vs. 59.4%, respectively, *P* < 0.001; stage IV, 6.3 vs. 30.1%, respectively, *P* < 0.001; stages I–IV, 17.7 vs. 55.9%, respectively, *P* < 0.001) (Figure 2).

### Liver Metastasis and High Alpha-Fetoprotein-Producing Levels Suggest Poor Prognosis in Patients With Alpha-Fetoprotein-Producing Adenocarcinoma From the Gastrointestinal Tract

Liver metastasis was found to be an independent risk factor for total survival in APA-GI patients after multivariate Cox regression analysis (hazard ratio 3.59, 95% confidence interval 1.28–10.02, *P* = 0.015) (Table 4). To investigate the impact of liver metastasis-free survival, we further conducted Cox multivariate regression analysis on the data, including the level of AFP. We found that AFP ≥ 200 ng/ml was an independent risk factor for the liver metastasis of APA-GI (hazard ratio 4.55, 95% confidence interval 1.39–14.87, *P* = 0.012) (Table 5).

**Table 4:** Regression analysis for prognostic risk factors of APA-GI.

**Variable**	**Single factor**		**Multiple factors**	
	**HR(95%CI)**	***P*–value**	**HR(95%CI)**	***P*-value**
Age, y		0.078		0.913
<60	1		1	
≥60	1.40 (0.96–2.05)		1.05 (0.42–2.62)	
Sex		0.075		0.551
Male	1		1	
Female	0.70 (0.47–1.04)		1.38 (0.48–3.94)	
AFP, ng/ml		0.005		0.877
<200	1		1	
≥200	1.67 (1.17–2.38)		0.93 (0.38–2.27)	
Primary site		0.241		
Stomach	1			
Large intestine	0.57 (0.32–1.02)			
Small intestine	0.66 (0.16–2.66)			
Esophagus	1.04 (0.14–7.45)			
Multiple primary sites	0.26 (0.04–1.86)			
Tumor size, cm		0.028		0.59
<5	1		1	
≥5	1.99 (1.08–3.65)		0.79 (0.34–1.83)	
Pathological type		0.660		
Adenocarcinoma	1			
Signet ring cell carcinoma	0.78 (0.44–1.37)			
Mucinous adenocarcinoma	0.88 (0.45–1.75)			
Differentiation		0.926		
Highly differentiated	1			
Moderately differentiated	0.90 (0.21–3.79)			
Poorly differentiated	0.84 (0.20–3.42)			
pT		0.056		0.96
1	1		1	
2	1.60 (0.32–7.98)		1.52 (0.15–15.72)	
3	2.68 (0.49–14.73)		1.95 (0.18–21.38)	
4	3.98 (0.96–16.50)		1.60 (0.18–14.01)	
pN		0.028		0.071
0	1		1	
1	2.54 (0.99–6.57)		2.80 (0.79–9.90)	
2+3	2.68 (1.29–5.56)		3.05 (1.16–8.06)	
TNM staging		<0.001		0.486
I-III	1		1	
IV	3.99 (2.69–5.90)		1.39 (0.55-3.53)	
Extrahepatic metastasis		0.031		0.127
No	1		1	
Yes	1.50 (1.04–2.16)		1.95 (0.83–4.60)	
Hepatic metastasis		<0.001		0.015
No	1		1	
Yes	2.79 (1.91–4.06)		3.59 (1.28–10.02)	
Lymphatic or/and blood vessel invasion		0.009		0.140
No	1		1	
Yes	2.30 (1.24–4.29)		2.04 (0.79–5.27)	
Nerve invasion		0.064		0.884
No	1		1	
Yes	1.76 (0.97–3.21)		0.92 (0.29–2.92)	
Hepatoid Adenocarcinoma^#^		0.102		0.228
No	1			
Yes	1.68 (0.90–3.12)			
AFP IHC		0.375		0.288
Low expression	1			
High expression	1.35 (0.70–2.62)			

# *73 surgical patients out of 100 patients receiving second pathological diagnosis were included*.

**Table 5:** Regression analysis for prognostic risk factors of APA-GI liver metastasis.

**Variable**	**Single factor**		**Multiple factors**	
	**HR(95%CI)**	***P*-value**	**HR(95%CI0)**	***P*-value**
Age, y		0.023		0.193
<60	1		1	
≥60	1.72 (1.08–2.74)		2.13 (0.68–6.65)	
Sex		0.021		0.123
Male	1		1	
Female	0.55 (0.33–0.91)		0.30 (0.07–1.38)	
Primary site		0.906		
Stomach	1			
Large intestine	0.89 (0.47–1.67)			
Other	0.86 (0.27–2.72)			
Tumor size, cm		0.010		0.231
<5	1		1	
≥5	3.47 (1.35–8.89)		1.96 (0.65–5.90)	
Pathological type		0.101		
Adenocarcinoma	1			
Signet ring cell carcinoma	0.50 (0.23–1.09)			
Mucinous adenocarcinoma	0.45 (0.14–1.44)			
Differentiation		0.741		
Highly differentiated	1			
Moderately differentiated	1.34 (0.18–9.94)			
Poorly differentiated	1.10 (0.15–8.00)			
pT		0.113		
1+2+3	1			
4	1.26 (0.95–1.68)			
pN		0.603		
0	1			
1	1.65 (0.56–4.85)			
2+3	1.10 (0.45–2.73)			
AFP, ng/ml		<0.001		0.012
<200	1		1	
≥200	2.24 (1.48–3.39)		4.55 (1.39–14.87)	
Lymphatic or/and blood vessel invasion		0.013		0.354
No	1		1	
Yes	3.31 (1.29–8.49)		1.69 (0.56–5.14)	
Nerve invasion		0.277		
No	1			
Yes	1.6 (0.69–3.73)			
Hepatoid Adenocarcinoma^#^		0.029		0.615
No	1		1	
Yes	2.60 (1.10–6.12)		0.75 (0.35–2.27)	
AFP IHC		0.626		
Low expression	1			
High expression	1.22 (0.54–2.77)			

# *73 surgical patients out of 100 patients receiving second pathological diagnosis were included*.

**Figure 3:**
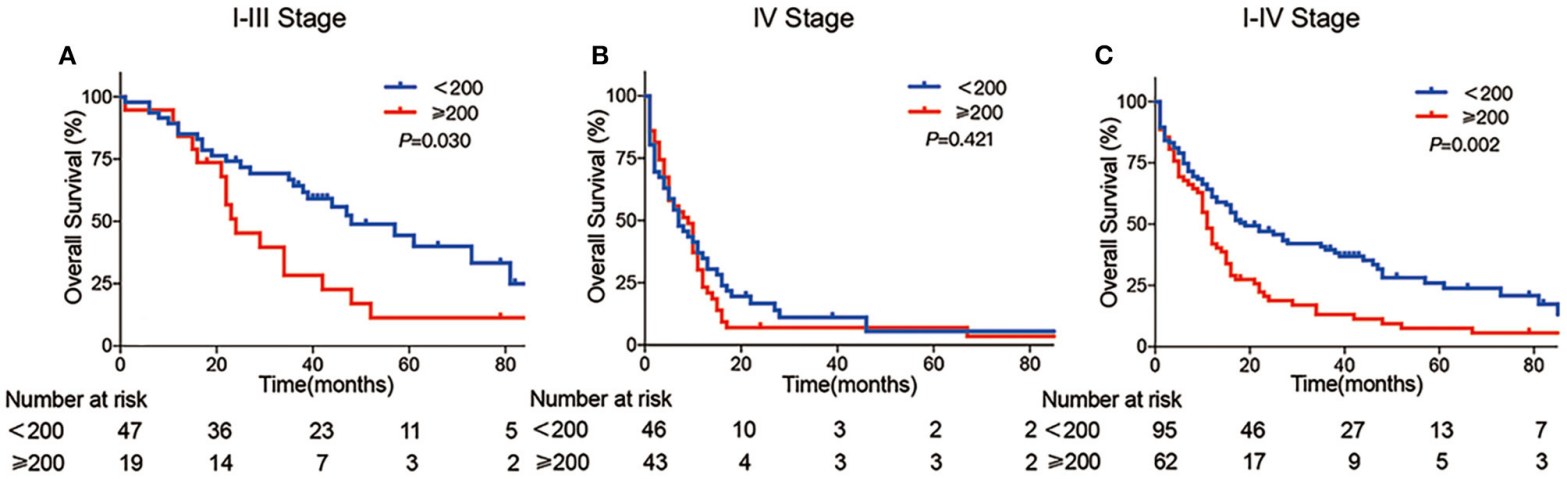
Kaplan–Meier curve of APA-GI compared by AFP level. Comparison of survival between APA-GI patients with AFP ≥ 200 ng/ml and AFP <200 ng/ml: **(A)** compare between stages I–III patients; **(B)** compare between stage IV patients; **(C)** compare between stages I–IV patients. Notably, two patients were unable to perform accurate staging due to the lack of medical imaging data. Therefore, they can only be classified as stages I–IV. IRS 0–1, 2–3, 4–8, and 9–12 was referred to negative, week positive, moderate positive and strong positive, respectively.

Kaplan–Meier curve that showed the prognosis of the AFP-H group was significantly worse than that of the AFP-L group. The 5-year survival of AFP ≥ 200 ng/ml was 7.5%, whereas it increased to 26.0% in the APA-GI patients with AFP <200 ng/ml (*P* = 0.002). The proportion of stage IV patients in the AFP-H and AFP-L groups were 71 and 50%, respectively. The prognosis of stages I–III patients with an AFP ≥ 200 ng/ml was worse than that of AFP <200 ng/ml (5-year survival: 11.3 vs. 44.4%, respectively, *P* = 0.030), whereas there was no statistical difference in the prognosis of stage IV patients (5-year survival: 3.5 vs. 5.6%, respectively, *P* = 0.421) (Figure 3).

## Discussion

APA is a rare kind of malignant tumor, which could originate from various organs, such as the lung, ovary, kidney, esophagus, stomach, small intestine, colorectal, and appendix (3, 10, 14–18). The present study focused on the clinicopathological features of APA-GI *via* a retrospective analysis. Data from SAHZU showed APA-GI was more likely to occur in middle-aged and older adults, and it could originate from the whole GI tract, especially in the stomach. The rate of tumor metastasis is high, and most patients were initially diagnosed with liver metastasis. The positive rate of AFP in APA-GI tumor tissue was 36.4%, and it was partly expressed in hepatoid differentiated tissue. The prognosis of APA-GI was significantly worse than CA-GI, and patients with AFP ≥ 200 ng/ml were even worse in APA-GI. Liver metastasis was an independent risk factor for the overall survival of APA-GI, and patients with AFP ≥ 200 ng/ml were more like to develop liver metastasis. Ren et al. had also previously investigated the clinicopathological features and prognosis of APA-CRC, especially the tumor location and survival, which was with our research. Additionally, they divided APA-CRC patients into three histologic types: the common adenocarcinoma type, mucinous adenocarcinoma type, and hepatoid type (3). However, the present research still had some progress. We provided a sufficient sample size with 177 APA-GIs to reach statistically significant conclusions. We included not only APA-CRC but also other GI sites' APA to obtain a full landscape of APA-GI. In addition, we also described several risk factors of liver metastasis and prognosis.

Most APA-GIs were low differentiation. The tumor cells were distributed in clusters or bands, and the stroma was rich in blood sinuses. HA was the main pathological feature of APA-GI. However, among 100 patients included, only three were reported HA in the original pathological diagnosis, whereas there were actually 34 HA patients after reviewing all HE sections. The main reasons for missed diagnosis of APA-GI may be as follows. First, HA is often intermixed within CA components, making the former difficult to recognize, and it leads to the missed diagnosis of APA-GI. Secondly, HA is not necessary to report in routine pathological diagnosis, making pathologists pay insufficient attention. Although HA is the major pathologic type of APA-GI, only 30% of HA cases have been reported within APA-GI in our center. Therefore, the serum AFP index is still essential for the diagnosis of APA-GI. The current diagnostic criteria for HA proposed by the World Health Organization relies on pathological morphology without the need for IHC evidence (World Health Organization Classification of Tumours, 5th Edition, Volume 1. Digestive System Tumours. Lyon: IARC Press, 2019) (19). Therefore, the present study asked two pathologists to independently review HE sections to confirm hepatoid differentiation without additional Hepa-1 and glypican-3 IHC. Additionally, the survival was similar between APA-GI with and without hepatoid differentiation, but both were worse than that of CA-GI. Therefore, using only hepatoid differentiation in the diagnosis of APA-GI will lead to missed diagnosis and inadequate understanding of prognosis. In previous studies, elevated serum AFP levels, positive AFP IHC, or HA detection was used as diagnostic criteria for APA-GI, but there is no standard criteria or consensus until now (6, 20–22). In our study, neither HA nor positive AFP IHC was the sufficient condition of APA-GI diagnosis, as only 52% of patients existed either HA or positive AFP IHC. For these patients, their prognoses were similar to that of APA-GI patients with only elevated serum AFP. Therefore, we suggested APA-GI should be regarded as one kind of disease, and its diagnosis depends on both serum AFP level and pathology. Also, pathologists should pay more attention to hepatoid differentiation, and there should be more molecular pathology research to construct the diagnostic criteria of APA-GI.

High metastasis rate and liver metastasis are the main characteristics of APA-GI at initial diagnosis. Many studies have found that 12–25% of patients with digestive tract adenocarcinoma have metastases after initial diagnosis (23, 24). Nearly 40–50% of patients have metastases during the whole course of the disease (24, 25). Among them, 4–14% gastric adenocarcinomas and 25% colorectal adenocarcinomas were initially diagnosed with liver metastasis, and approximately 50% of the patients with digestive adenocarcinoma eventually developed liver metastasis (26, 27). Our study found that in APA-GI, up to 58.2% of patients metastasized after the initial diagnosis, and the final metastasis rate reached 81.9%. Among APA-GI, patients with liver metastasis at initial diagnosis were significantly more than those with CA-GI. Additionally, we performed a subgroup analysis by serum AFP with a bound of 200 ng/ml. Consistent with previous studies, the AFP-H group was characterized by a higher rate of stage IV and liver metastasis, a larger tumor size, and a higher proportion of HA (5, 28, 29).

The prognosis of APA-GI was significantly worse than that of CA-GI. However, we did not observe a difference between stages I–III gastric and CRC patients, which may be related to 95.5% of stages I–III patients who underwent surgery. Early diagnosis of APA-GI and radical surgery may be an important treatment to improve prognosis. When APA-GI progressed to stage IV, the 5-year survival was only 6.3%. We hypothesized that stage IV APA-GI patients were less sensitive to 5-Fu-based chemotherapy, similar to the 8.15% response rate of primary liver cancer to chemotherapy (30). Because of selection bias and recall bias in retrospective studies, the results of multivariate analyses varied among previous reports. Shoji and Feng et al. found that liver metastasis was the only independent prognostic factor for APA-GI (6, 31), whereas in other studies, TNM staging, serum AFP level, patient age, peritoneal seeding, lymph node metastasis, vascular invasion, Lauren classification, and AFP IHC results all became independent prognostic factors for APA-GI (29, 32). Our study showed that patients with high AFP levels had a poor prognosis, whereas Cox regression analysis showed that liver metastasis was the only independent risk factor for APA-GI. We speculated that it might be due to the strong influence of liver metastasis on prognosis, thus masking the influence of high AFP level on prognosis. There were still several limitations in the present research. AFP has been reported not only in APA but also in yolk-sac tumors or some neuroendocrine tumors (1, 33, 34). We asked two pathologists to recheck all HE sections and did not found any yolk sac tumor-like area or neuroendocrine component. However, for the other 77 patients without HE sections, we were unable to clarify whether AFP was produced only by APA. Whether APA-GI is homogenous or heterogenous could not be concluded yet. Wang et al. had investigated the molecular features of HA of the stomach but showed limited homogenous (13). Besides, HA is theoretically not exactly the same as APA-GI. In future studies, we need more data from multiple centers to further study the clinicopathological characteristics of APA-GI, and basic research should be encouraged to investigate the pathogenesis of APA-GI.

## Conclusion

The present study was the first research to systemically explored clinicopathological characteristics of APA-GI. APA-GI occurs through the whole GI tract with a significantly worse prognosis than CA-GI. APA-GI should be regarded as one kind of disease for its similar clinicopathological characteristics within patients.

## Data Availability Statement

The raw data supporting the conclusions of this article will be made available by the authors, without undue reservation.

## Author Contributions

X-XK, X-LL, and X-MX wrote the first draft of the manuscript. All authors commented on previous versions of the manuscript. All authors contributed to the study's conception and design. All authors performed material preparation, data collection, and analysis. All authors commented on previous versions of the manuscript. All authors read and approved the final manuscript.

## Conflict of Interest

The authors declare that the research was conducted in the absence of any commercial or financial relationships that could be construed as a potential conflict of interest.

## Supplementary Material

Supplementary Figure 1 Kaplan–Meier curve of HA positive and HA negative within APA-GI Comparison of survival between HA positive and HA negative within 73 APA-GI patients who received operation.

Supplementary Figure 2 Typical immunohistochemical staining images of APA-GI a, Negative; b, Weakly positive; c, Moderately positive; d, Strongly positive.

Supplementary Figure 3 Kaplan–Meier curve of AFP-/HA- between the other patients within APA-GI. Comparison of survival between AFP-/HA- and the other patients within 73 APA-GI patients who received operation.
