# Supplementary figures and images for: The Clinicopathological Characteristics of Alpha-Fetoprotein-Producing Adenocarcinoma of the Gastrointestinal Tract—A Single-Center Retrospective Study
Source: Front Oncol. 2021 Apr 29;11:635537. doi: 10.3389/fonc.2021.635537 (PMC8118715; doi:10.3389/fonc.2021.635537)

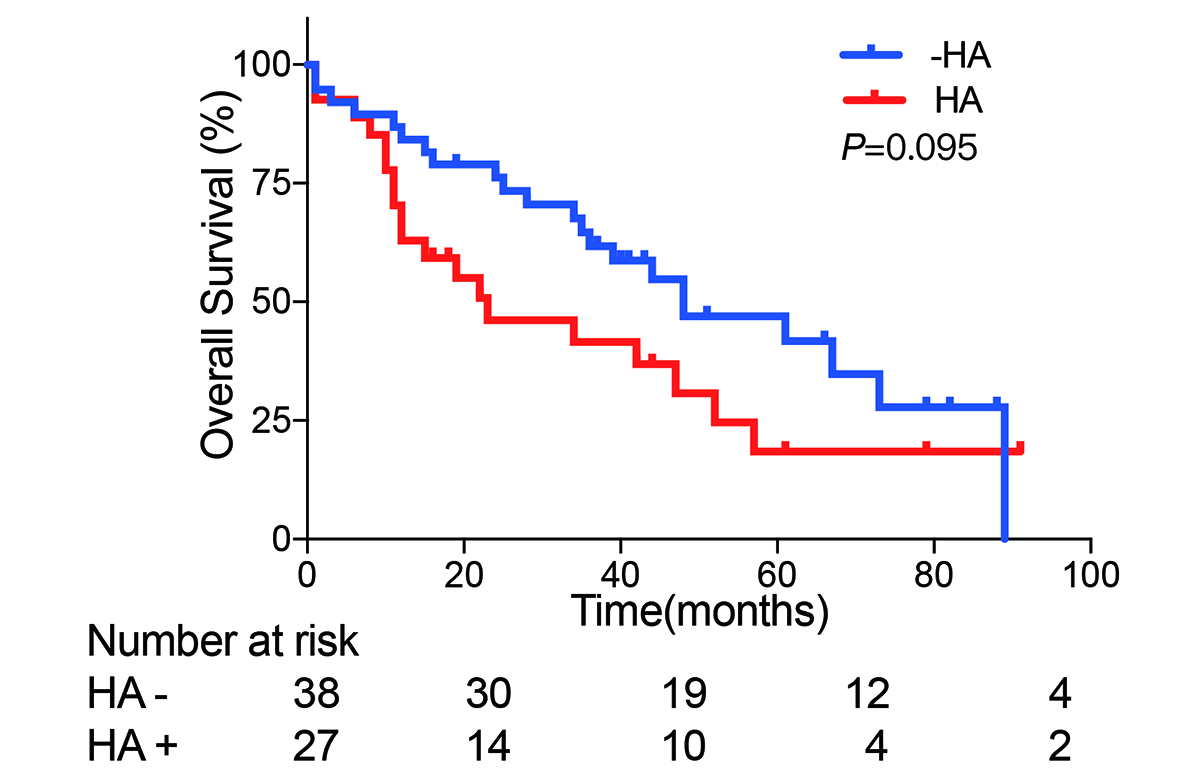

Supplement: Supplementary Figure 1 — Kaplan–Meier curve of HA positive and HA negative within APA-GI Comparison of survival between HA positive and HA negative within 73 APA-GI patients who received operation. [file Image_1.TIF]

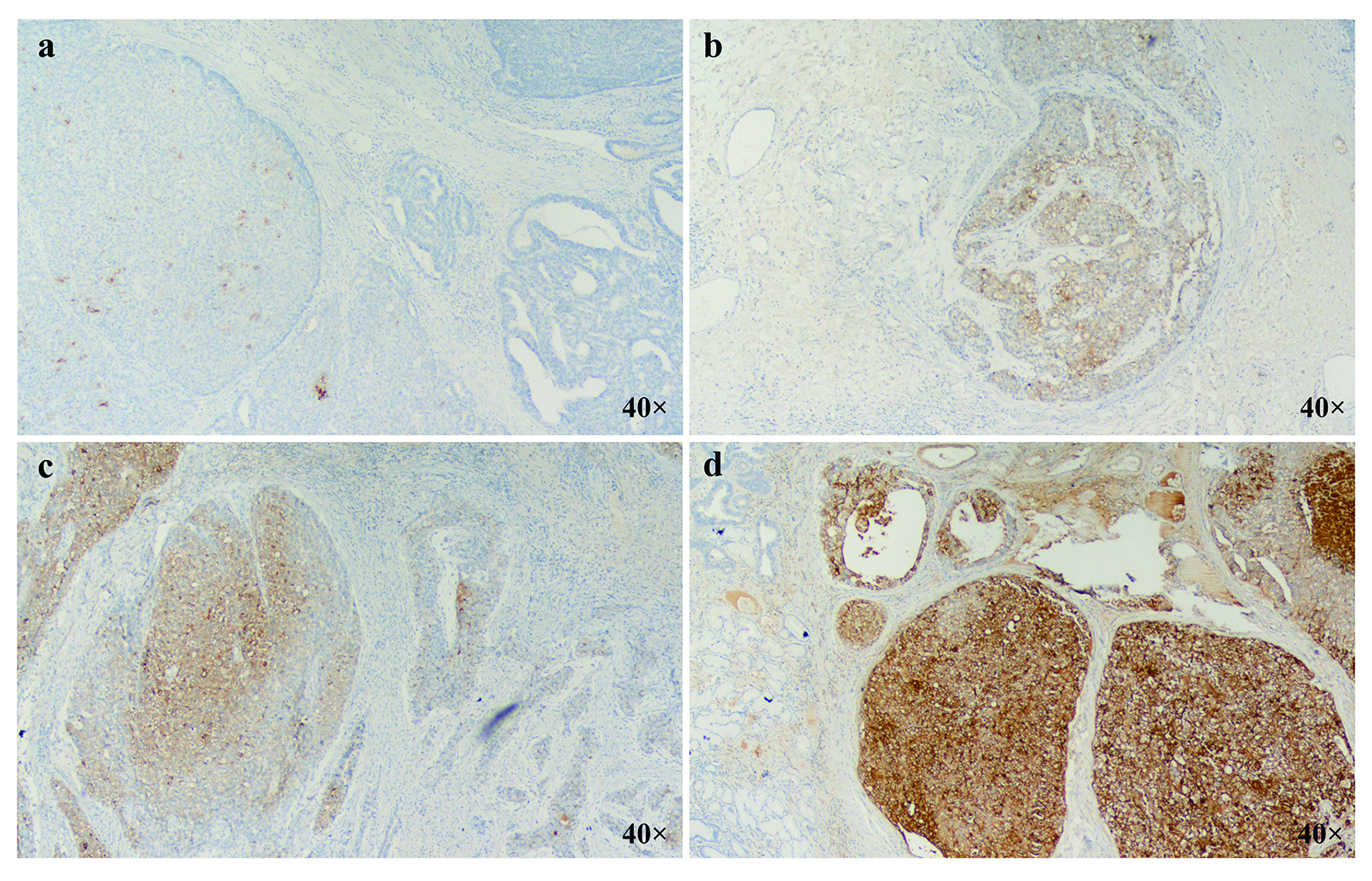

Supplement: Supplementary Figure 2 — Typical immunohistochemical staining images of APA-GI a, Negative; b, Weakly positive; c, Moderately positive; d, Strongly positive. [file Image_2.TIF]

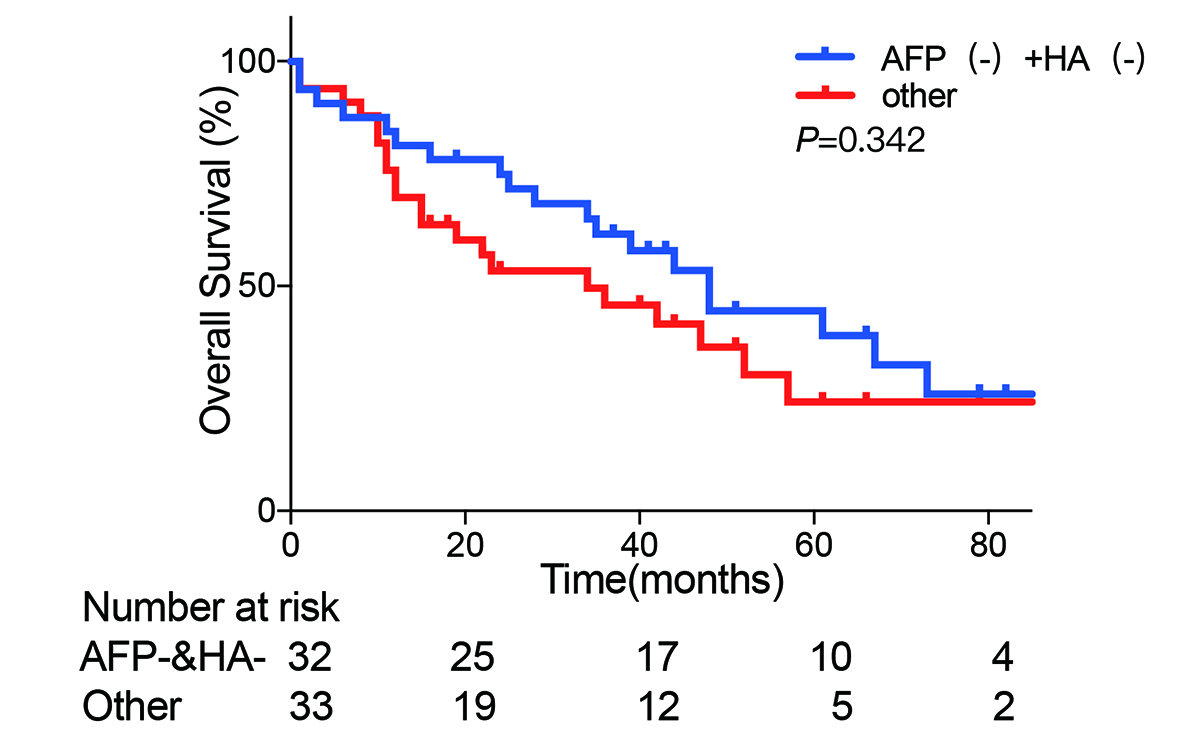

Supplement: Supplementary Figure 3 — Kaplan–Meier curve of AFP-/HA- between the other patients within APA-GI. Comparison of survival between AFP-/HA- and the other patients within 73 APA-GI patients who received operation. [file Image_3.TIF]
